# Moving From the Epidemiology to the Treatment of Respiratory Failure in Patients With Cardiogenic Shock

**DOI:** 10.1016/j.jacadv.2025.101970

**Published:** 2025-07-23

**Authors:** P. Elliott Miller, Omar El Charif, Mark Jacobs

**Affiliations:** a Section of Cardiovascular Medicine, Yale School of Medicine, New Haven, Connecticut, USA; b Department of Critical Care Medicine, Montefiore Medical Center, Bronx, New York, USA

**Keywords:** cardiogenic shock, invasive mechanical ventilation, respiratory failure

**Figure:**
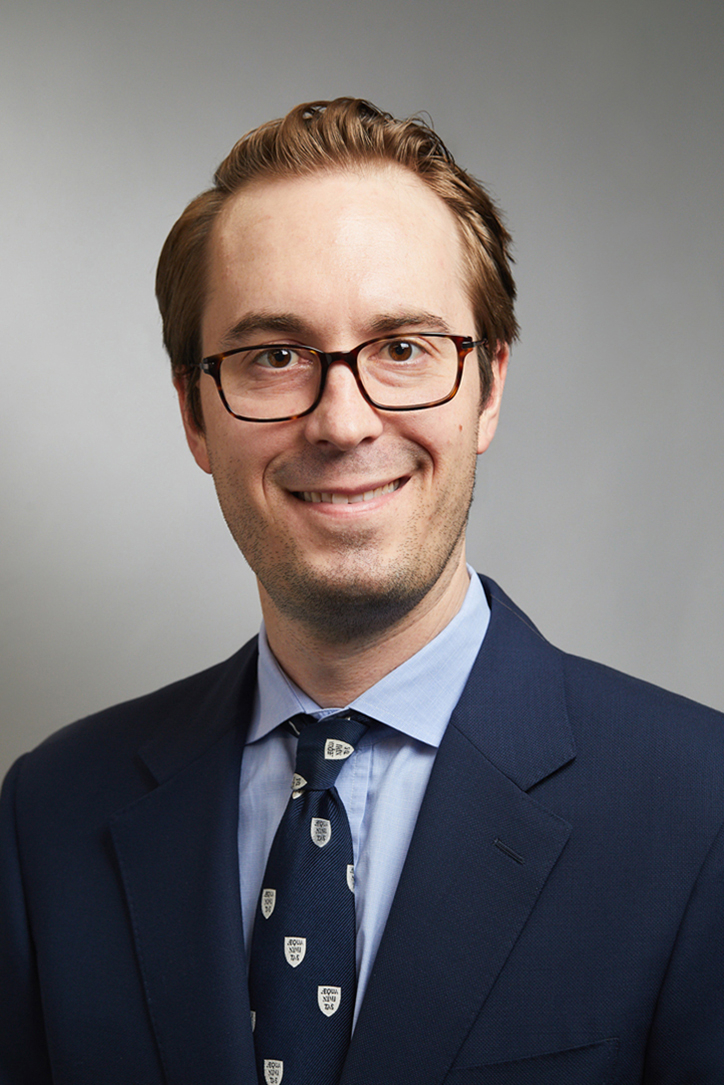

“Medicine is a science of uncertainty and an art of probability.”

—William Osler^1^

As the modern cardiac intensive care unit (CICU) has evolved, so has the case mix and indications for CICU admission. In particular, respiratory failure is now the most common reason for admission to the CICU,^2^ and upward of 80% of patients with cardiogenic shock require invasive mechanical ventilation (IMV).^3^ As expected, patients with cardiovascular disease requiring respiratory support have significantly worse outcomes.^4^, ^5^, ^6^ However, data beyond the association between IMV and clinical outcomes, including indications for respiratory support, ventilator settings, induction/sedation practices, and extubation strategies, is sparse at best. Numerous expert reviews have been published, but the evidence base remains limited,^7^^,^^8^ especially as patients with cardiovascular disease are frequently excluded from intensive care studies investigating respiratory failure.^9^ Given these important gaps in the literature, developing management strategies for patients with cardiovascular disease and respiratory failure has been identified as a key research priority.^10^^,^^11^

In this issue of *JACC: Advances*, Grubb et al^12^ detail their single-center experience, including 104 patients with Society for Cardiovascular Angiography & Interventions stage C-E cardiogenic shock from 2017 to 2023 who required IMV. To be included, patients were required to have undergone right heart catheterization and either receive vasoactive medications and/or mechanical circulatory support (MCS). The cohort included patients with cardiogenic shock from both decompensated heart failure (65%) and acute myocardial infarction (35%). Notably, the patients included in this study were very critically ill, as evidenced by a median admission lactate of 4.7 mmol/L, a high rate of MCS during IMV (49%), advanced shock stage (half at Society for Cardiovascular Angiography & Interventions shock stage E at the time of intubation), and an in-hospital mortality of 41%. While a sample size of 104 patients may seem small, we would suggest it represents a large number of the sickest patients admitted to any CICU.

The authors present a treasure trove of detailed information that, to date, can only be captured in a comprehensive, single-center study due to limitations of currently available cardiogenic shock databases/registries. The largest proportion of patients in their cohort were intubated for ongoing cardiac arrest (37%), with the second most common etiology being hypoxic respiratory failure (32%). The most frequently used induction agents were rocuronium and etomidate, likely reflecting attempts to limit hemodynamic perturbations in an unstable patient population. Despite attempts at hemodynamically neutral agents, approximately 40% of patients had a peri-intubation complication, most commonly early hypotension requiring hemodynamic intervention. Volume control mode was used in approximately 90% of patients, and the median tidal volume was 7 cc/kg. Although the majority of patients were on minimal ventilator settings (minimal oxygen and positive end-expiratory pressure levels) by 24 hours, the median IMV support was 4.8 days.

Equally as important, the authors detail their experience with liberating these critically ill patients from IMV. Despite minimal ventilator settings at 24 hours, only 26% and 22% of patients underwent a spontaneous awakening trial (SAT) and spontaneous breathing trial (SBT) within 48 hours of intubation, respectively. The most common reasons cited for delayed SAT/SBTs were hemodynamic instability and tachycardia/arrhythmia, and only 1 patient was extubated with MCS in place.

Being a single-center study, there are certainly site-specific practice patterns that may not be generalizable to other institutions. The authors report a co-management model between a pulmonary critical care consultant and the primary cardiology team, but further details on how responsibilities were delineated and how disagreements were settled are not included. At 73%, benzodiazepine use was high, but there is little published data to use as a benchmark, and per hospital policy, dexmedetomidine was restricted to within 48 hours of expected extubation. It is also important to note that the most common etiology for intubation was ongoing cardiac arrest, which is inherently a very different group than those requiring IMV for respiratory failure, whether that be for hypoxia, hypercarbia, mixed hypoxia/hypercarbia, or increased work of breathing. The fact that most patients were on minimal ventilator settings by 24 hours further supports that many patients were less likely intubated for primary respiratory failure. Reintubation rates were also high: 14% at 48 hours, compared to multicenter CICU data from the Critical Care Cardiology Trials Network (CCCTN), which found reintubation rates of 7.6%, albeit in a mixed patient population and not just patients with cardiogenic shock.^2^

Despite these limitations and potentially center-specific nuances, the authors should be congratulated for an incredibly well-phenotyped patient population and important study. As the authors state, this study is not intended or powered to compare specific therapies but to detail current practice patterns of an understudied but critical aspect of cardiogenic shock management. Their work will provide an important reference for further studies on respiratory failure in patients with cardiogenic shock and likely inspire future research.

Numerous questions are easily generated from their study. What induction agent should we use for patients with cardiogenic shock? What is the best sedation strategy for this patient population? Does it matter depending on the etiology of shock? There is some recently published hypothesis-generating data for both induction agents and sedation in patients with acute myocardial infarction,^13^^,^^14^ but both require validation and more research before changing practice. How do we develop CICU-specific SAT/SBT criteria? Are we delaying extubation by waiting for removal of femoral access MCS? While heart failure is a known risk factor for extubation failure, should all patients be extubated to respiratory support? Would certain patients benefit from noninvasive positive pressure ventilation or high-flow nasal cannula? Other important questions involve the differences in practice patterns and outcomes among different subpopulations included in this study, most notably acute myocardial infarction vs heart failure patients and cardiac arrest vs noncardiac arrest patients. Furthermore, additional variability in IMV management is expected in isolated left ventricular vs isolated right ventricular vs biventricular shock.

Finally, it is expected that institution-specific factors introduce a great deal of variability in overall practice patterns, as has been demonstrated repeatedly in CCCTN literature. Therefore, for many of these questions, the next steps should include characterization across multiple centers, potentially through the creation of registries specific to respiratory failure in the CICU. Embedding modules specific to respiratory status/IMV in established registries such as CCCTN or the Cardiogenic Shock Working Group would offer a valuable opportunity for multicenter analysis but may lack the granular insights from a respiratory failure-specific registry. To be sure, dedicated CICU research is sorely needed to move the management of respiratory failure in the acute cardiac patient from an art toward more of a science.

## Funding support and author disclosures

The authors have reported that they have no relationships relevant to the contents of this paper to disclose.
